# Sparse-view synchrotron X-ray tomographic reconstruction with learning-based sinogram synthesis

**DOI:** 10.1107/S1600577523008032

**Published:** 2023-10-17

**Authors:** Chang-Chieh Cheng, Ming-Hsuan Chiang, Chao-Hong Yeh, Tsung-Tse Lee, Yu-Tai Ching, Yeukuang Hwu, Ann-Shyn Chiang

**Affiliations:** a Information Technology Service Center, National Yang Ming Chiao Tung University, 1001 University Road, Hsinchu, Taiwan; b Department of Computer Science, National Yang Ming Chiao Tung University, 1001 University Road, Hsinchu, Taiwan; c Institute of Data Science and Engineering, National Yang Ming Chiao Tung University, 1001 University Road, Hsinchu, Taiwan; d Institute of Physics, Academia Sinica, 128 Academia Road, Nankang, Taipei, Taiwan; e Brain Research Center, National Tsing Hua University, Hsinchu 30013, Taiwan; Australian Synchrotron, Australia

**Keywords:** sparse-view computed tomography, synchrotron X-ray computed tomography, deep learning, view interpolation, sinogram synthesis

## Abstract

Synchrotron radiation can be used as a light source in X-ray microscopy to acquire a high-resolution image of a microscale object for tomography. However, numerous projections must be captured for a high-quality tomographic image to be reconstructed; thus, image acquisition is time consuming. Such dense imaging is not only expensive and time consuming but also results in the target receiving a large dose of radiation. To resolve these problems, sparse acquisition techniques have been proposed; however, the generated images often have many artefacts and are noisy. In this study, a deep-learning-based approach is proposed for the tomographic reconstruction of sparse-view projections that are acquired with a synchrotron light source; this approach proceeds as follows. A convolutional neural network (CNN) is used to first interpolate sparse X-ray projections and then synthesize a sufficiently large set of images to produce a sinogram. After the sinogram is constructed, a second CNN is used for error correction. In experiments, this method successfully produced high-quality tomography images from sparse-view projections for two data sets comprising *Drosophila* and mouse tomography images. However, the initial results for the smaller mouse data set were poor; therefore, transfer learning was used to apply the *Drosophila* model to the mouse data set, greatly improving the quality of the reconstructed sinogram. The method could be used to achieve high-quality tomography while reducing the radiation dose to imaging subjects and the imaging time and cost.

## Abstract 2

This article proposes a deep-learning-based approach for synchrotron X-ray computed tomography with sparse-view projections. The experimental results indicate that tomographic images can be reconstructed by 75 X-ray projections without obvious streak artefacts and noise.

## 1. Introduction

Synchrotron X-ray computed tomography (SXCT) can be applied to acquire tomographic images for microscale or nanoscale objects (Stampanoni *et al.*, 2002 ▸), and has been used for both industrial applications (Lo *et al.*, 2007 ▸) and biology research (Chien *et al.*, 2012 ▸).

The Nyquist–Shannon sampling theorem (Shannon, 1949 ▸) states that, to accurately reconstruct a signal or image, the sampling rate must be at least twice the highest-frequency component of the signal. In tomography, this translates to the requirement that the number of projections should be at least twice the number of pixels in the direction of rotation to ensure that the object is adequately sampled and that the reconstructed volume is free from aliasing artefacts. The Crowther criteron (Jacobsen, 2018 ▸) indicates that the number of projection views should be *N*_θ_ = (π/2) *N*_t_ for a tomographic image of 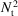 pixels to be constructed. However, the number of projection views should be minimized to prevent the target object from receiving an excessive dose of radiation. Numerous low-dose computed tomography techniques have been developed for medical imaging (Zhu *et al.*, 2004 ▸; Rampinelli *et al.*, 2012 ▸). One such method is sparse-view computed tomography (SVCT) (Kudo *et al.*, 2013 ▸; Labriet *et al.*, 2018 ▸; Liu *et al.*, 2020 ▸), in which the imaging dose can be decreased by reducing the number of projection views. In SVCT, the aim is to use fewer than *N*_θ_ projection views to reconstruct an image of 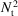 pixels without visible artefacts or noise. However, directional reconstruction methods for sparse-view projections, such as the filtered back-projection (FBP) algorithm and the simultaneous algebraic reconstruction technique (Kak & Slaney, 2001 ▸), may produce streak artefacts. For example, visible artefacts and noise are produced if an image of 512^2^ pixels is reconstructed from fewer than 100 views.

**Figure 1:**
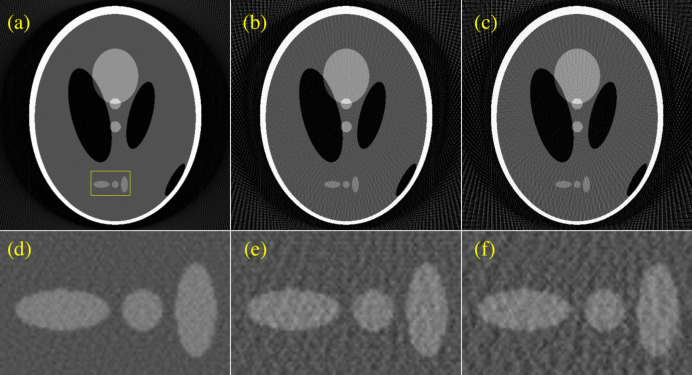
FBP results for (*a*) 180, (*b*) 90 and (*c*) 75 projection views. (*d*), (*e*) and (*f*) Magnifications of the boxed region in (*a*) for (*a*), (*b*) and (*c*), respectively.

Figures 1 ▸(*a*), 1 ▸(*b*) and 1 ▸(*c*) present 512 × 512 pixel images produced by FBP from 180, 90 and 75 projection views, respectively. Figures 1 ▸(*d*), 1 ▸(*e*) and 1 ▸(*f*) display magnifications of the region bounded by the yellow rectangle in each image. Numerous streak artefacts and noise are apparent in the 75-view image in Fig. 1 ▸(*f*). Several iterative algorithms have been developed to improve the quality of SVCT, such as methods based on total variation (Sidky & Pan, 2008 ▸), non-local means (Chen *et al.*, 2009 ▸) and dictionary learning (Xu *et al.*, 2012 ▸; Li *et al.*, 2014 ▸). Although iterative algorithms can significantly reduce the artefacts and noise in SVCT, they may have an overly high computational cost. SVCT can also be used with interpolation-based methods to synthesize sinograms (Brooks *et al.*, 1978 ▸). Improved interpolation-based methods based on partial differential equations (Kostler *et al.*, 2006 ▸) or principal component analysis (Chen *et al.*, 2004 ▸) have also been proposed.

Moreover, deep-learning methods for SVCT with parallel and fan-beam projection geometries have been recently developed. Fu *et al.* (2020 ▸) proposed a convolutional neural network (CNN) for completing fragmentary differential phase-contrast sinograms. Chen *et al.* (2017 ▸) proposed a residual encoder–decoder CNN for removing artefacts in tomographic images. Jin *et al.* (2017 ▸) developed a network combining U-Net (Ronneberger *et al.*, 2015 ▸) with a residual network to remove artefacts while preserving the image structure. Lee *et al.* (2019 ▸) used a residual-based U-Net (RU-Net) to synthesize sinograms from sparse-view projections.

In cone-beam projection (Kak & Slaney, 2001 ▸; Scarfe *et al.*, 2006 ▸; Kumar *et al.*, 2015 ▸), which is a three-dimensional tomography technique, a point light source is used to acquire a series of two-dimensional X-ray projections of a detection plane from various views; these images can be used to synthesize three-dimensional tomographic volume data. Two deep-learning approaches have been proposed for SVCT with cone-beam projection. Hu *et al.* (2021 ▸) used two U-Nets to separately enhance interpolated projections and denoise reconstructed images, and Chao *et al.* (2022 ▸) proposed two encoder–decoder CNNs for separately interpolating projections and improving the quality of reconstructed images.

Although the X-rays are emitted from a point light source in SXCT, the emission of these X-rays can be considered an instance of parallel-beam projection because the object is typically on the millimetre or nanometre scale whereas the light source is several metres from the detector (Cheng *et al.*, 2014 ▸). Therefore, aliasing artefacts caused by beam divergence (Schulze *et al.*, 2011 ▸) are negligible. In this work, we propose a deep-learning method for SXCT with sparse-view projections. First, the sparse data were augmented by synthesizing a sequence of two-dimensional X-ray projections for the missing view angles with a CNN-based video-frame interpolation method. Subsequently, the synthesized images were transformed to sinograms and the method proposed by Lee *et al.* (2019 ▸) was employed to correct errors. Data sets from mice and *Drosophila* were collected to train and validate the proposed model.

## 2. Methods

**Figure 2:**
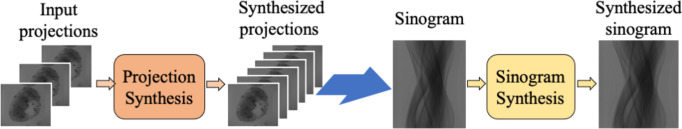
An overview of our synthesis method. First, the input sparse projections are interpolated to synthesize new images for the missing views. This mixed data set is then used to produce a sinogram. Finally, an error-correction method is applied to this sinogram.

Figure 2 ▸ presents the steps of the proposed method. First, the input projections are used to synthesize the missing view angles. Sinogram synthesis then corrects the errors of sinograms transformed from the synthesized projections. The horizontal and vertical axes of each sinogram represent the X-ray detector locations and the projection angles, respectively. For all image sets, the projection-angle range was at least 180°. The following two subsections describe the details of the projection- and sinogram-synthesis methods.

### 2.1. Projection synthesis

For projection synthesis, we adopted a CNN-based video-frame interpolation method to produce two-dimensional projection data for the missing view angles. Video-frame interpolation is a technique of increasing video frame rates by smoothing the transitions between two video frames. Numerous methods of video-frame interpolation have been proposed, such as a method of smoothing the interpolated frame with pixel-domain distributed video coding (Ascenso *et al.*, 2005 ▸) and a phase-based method in which motion is expressed as the phase shift of corresponding video-frame pixels (Meyer *et al.*, 2015 ▸). However, the interpolated frames produced by these methods are blurry and have artefacts. In recent years, several CNN-based video-frame interpolation methods have been proposed. Liu *et al.* (2017 ▸) proposed deep voxel flow (DVF), an end-to-end fully differentiable network for video-frame interpolation. They designed a convolutional encoder–decoder network to estimate the optical flow between input frames and used it to warp the input frames, producing interpolated frames. Although DVF does not produce blurry images, artefacts still remain. To improve the performance of DVF, Liu *et al.* (2019 ▸) proposed *CyclicGen*. *CyclicGen* uses a cycle-consistency loss function to ensure that the interpolated frames can be used to reconstruct the input frames without large errors.

**Figure 3:**
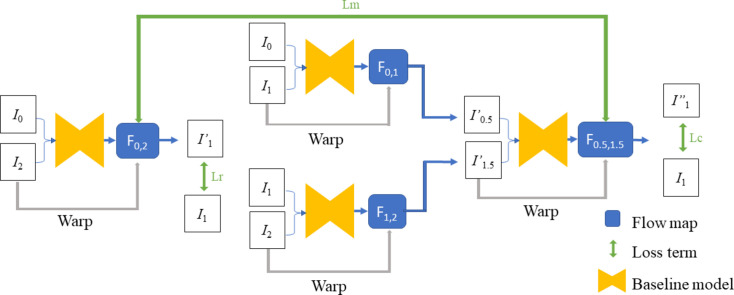
The structure of *CyclicGen*. First, *I*_0_, *I*_1_ and *I*_2_ are used to produce 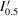 and 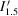 ; these are then used to produce 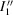 with minimal difference from *I*_1_.

Figure 3 ▸ depicts the architecture of *CyclicGen*. Each baseline model is a pretrained CNN model that produces a flow map *F*_*a*,*b*_ of the input frames *I*_*a*_ and *I*_*b*_. This flow map can then be used to generate an warped frame 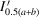 . Let *I*_*t*_ be the video frame taken for time *t*. *CyclicGen* first combines three video frames, *I*_0_, *I*_1_ and *I*_2_, to synthesize frames 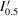 and 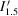 . Subsequently, *CyclicGen* combines 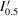 and 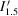 to synthesize 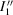 such that the difference between *I*_1_ and 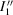 is minimized. In the figure, *L*_r_, *L*_c_ and *L*_m_ indicate the the loss functions of *CyclicGen*; these are the reconstruction loss, cycle-consistency loss and motion-linearity loss, respectively. *CyclicGen* also implements edge-guided training (Xie & Tu, 2015 ▸), which preserves the edge structure for better results.

We used *CyclicGen* to synthesize a series of two-dimensional projections instead of directly interpolating a sinogram. Because the projection-angle intervals for a series of two-dimensional X-ray projections for tomography are uniform, they can be considered to be a series of video frames; *CyclicGen* can then be used to increase the frame rate. For training, three consecutive two-dimensional projection frames composed a single training instance; the first and third projection frames were the input, and the second projection was the target.

### 2.2. Sinogram synthesis

**Figure 4:**
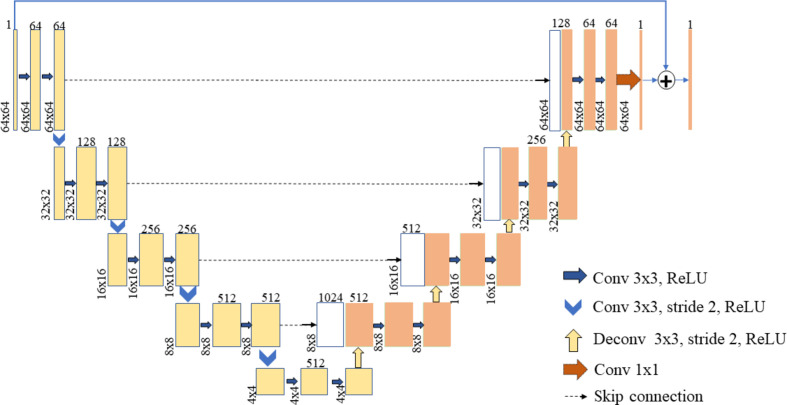
The structure of RU-Net in the sinogram-synthesis method. Each maximum pooling layer in U-Net was replaced by a convolutional block, and residual learning was added.

Lee *et al.* (2019 ▸) proposed RU-Net to enhance the performance of U-Net and used it to correct errors in sinograms synthesized from scarce data. Figure 4 ▸ displays the structure of RU-Net. The U-Net structure is based on the encoder–decoder model (Cho *et al.*, 2014 ▸). Each level of the encoder and decoder comprises a series of convolutional (conv) blocks and rectified linear units (ReLU). At each encoder level from top to bottom, the data size is halved by a convolutional block with a stride-2 kernel; these data are then input for the next level. Similarly, at each decoder level from bottom to top, a deconvolutional block (deconv) with a stride-2 kernal doubles the size of the output from the previous level. Skip connections provide the output of each encoder as input for the decoder on the same level. The technique of residual learning (He *et al.*, 2016 ▸) is applied in RU-Net. In Fig. 4 ▸, the final output is the sum of the original input and the output data of the last decoder level.

Lee *et al.* (2019 ▸) used simple linear interpolation to compensate for missing views in sparse input data; this could cause the details and edges to be blurred. To overcome this problem, we used *CyclicGen* to compensate for the missing projections before synthesizing the sinogram. The results presented in Section 3 demonstrate that the reconstruction quality of the proposed method is superior to that of the method formulated by Lee *et al.* (2019 ▸).

### 2.3. Training

We trained the learning models for the projection synthesis and sinogram synthesis with an ℓ^1^ norm loss function defined as follows: 

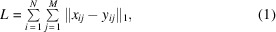

where *N* is the number of data instances, *M* is the number of patches, and *x*_*i*,*j*_ and *y*_*i*,*j*_ are the *j*th patches cropped from the *i*th input and target, respectively. For the projection-synthesis model (*CyclicGen*), the input and target were selected from a set of two-dimensional projection images with sufficient projection views. For the sinogram-synthesis model, the input was a sinogram produced by *CyclicGen* and the target was the ground-truth sinogram. We averaged the overlapping regions to stitch any two adjacent patches; the size of the overlap was half a patch.

## 3. Results

The proposed method was implemented in Python 3.7 and *TensorFlow* 2.5. A computer equipped with an Intel i7-12700 CPU, 64 GB of RAM and a NVIDIA RTX 4090 GPU was used to train the learning models. Two data sets obtained from biological experiments on *Drosophila* and mice were used to verify the proposed method (Stampfl *et al.*, 2023 ▸). The data sets were provided by the NanoX Laboratory, Institute of Physics, Academia Sinica (https://www.nanoxlab.org). All X-ray images were acquired with a light source from the Pohang Accelerator Laboratory. The beam flux and peak energy were within 10^7^–10^9^ photons s^−1^ mm^−2^ (150 mA) and 23–50 keV, respectively. The exposure time was 1 s per frame. The detector array had 512 × 512 elements and the pixel size was 1.875 µm. The *Drosophila* data set comprised 57 X-ray image sets of *Drosophila* brains and the mouse data set comprised 17 X-ray image sets of mouse brains. Each X-ray image set comprised 600 X-ray images with a projection-angle interval of 0.3°; the sizes of each X-ray image and sinogram were 512 × 512 and 600 × 512 pixels for the *Drosophila* and mouse sets, respectively. Each X-ray image and sinogram was sliced into 225 and 375 overlapping patches with sizes of 64 × 64 and 48 × 64 pixels, respectively. For model training, we randomly chose 49 and 13 image sets from the *Drosophila* and mouse sets, respectively. The remaining images composed the test data sets.

To simulate a sparse-view projection, we uniformly selected 75 X-ray images from each X-ray image, effectively increasing the projection-angle interval for each input to 2.4°.

The following subsections detail the experiments and results.

### 3.1. *Drosophila* data set

**Figure 5:**
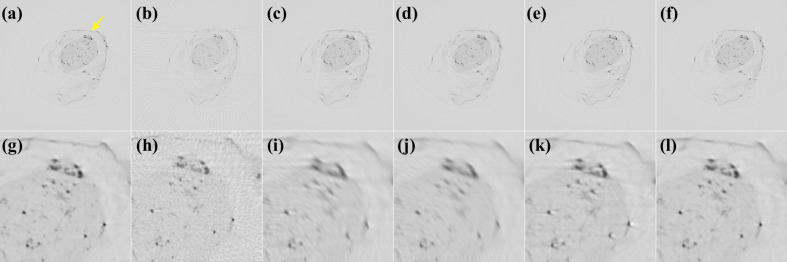
Tomographic images of *Drosophila*. (*a*) The ground-truth image and images reconstructed from 75 X-ray projection views (*b*) directly or with synthesis by (*c*) bicubic interpolation, (*d*) *CyclicGen*, (*e*) bicubic interpolation with RU-Net, or (*f*) *CyclicGen* with RU-Net. (*g*)–(*l*) Magnifications of the region indicated by a yellow arrow in (*a*) for the corresponding figures in the first row.

We first trained *CyclicGen* on the *Drosophila* data set for 37 h. Figure 5 ▸ presents the tomographic images reconstructed by FBP using the *Drosophila* X-ray projections; the second row displays enlargements of the region indicated by a yellow arrow in Fig. 5 ▸(*a*) for the corresponding image above it. Figure 5 ▸(*a*) presents the ground truth reconstructed from 600 real projections; each dark spot represents the cross section of a brain neuron stained with Golgi’s method (Chen *et al.*, 2021 ▸). Figure 5 ▸(*b*) presents the image directly reconstructed from 75 projections, and Figs. 5 ▸(*c*) and 5 ▸(*d*) present the images reconstructed from the projections synthesized by using bicubic interpolation and *CyclicGen*, respectively, to increase the number of projection views from 75 to 600. The *CyclicGen* image still had numerous artefacts and noise because the characteristics of the compositing sine waves were not considered for the *CyclicGen* synthesis.

**Figure 6:**
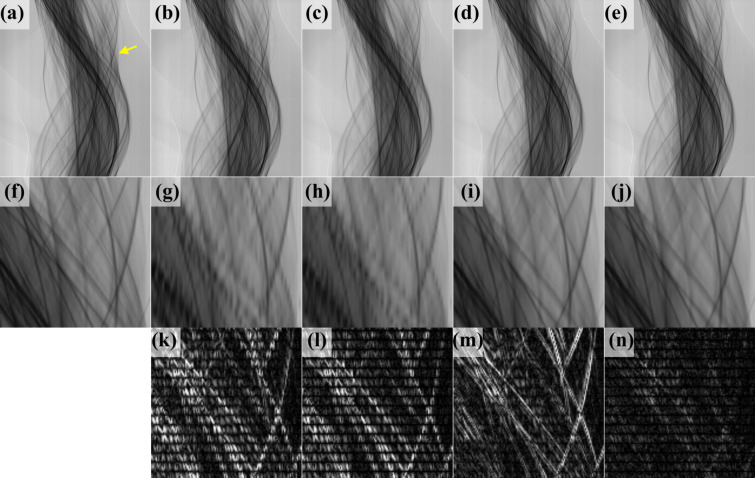
*Drosophila* sinograms. (*a*), (*b*), (*c*), (*d*) and (*e*) Sinograms corresponding to the images in Figs. 5 ▸(*a*), 5 ▸(*c*), 5 ▸(*d*), 5 ▸(*e*) and 5 ▸(*f*), respectively. (*f*)–(*j*) Magnifications of the region indicated by a yellow arrow in (*a*) for the corresponding figures in the first row. (*k*)–(*n*) Absolute differences between the ground truth (*f*) and the corresponding images on the second row. Pixels with higher brightness indicate larger errors.

Figures 6 ▸(*a*), 6 ▸(*b*), 6 ▸(*c*), 6 ▸(*d*) and 6 ▸(*e*) display the sinograms for Figs. 5 ▸(*a*), 5 ▸(*c*), 5 ▸(*d*), 5 ▸(*e*) and 5 ▸(*f*), respectively; the vertical and horizontal axes represent the projection angles and location of the X-ray detectors, respectively. The second row of Fig. 6 ▸ displays enlargements of the region indicated by a yellow arrow in Fig. 6 ▸(*a*) for the corresponding first-row images. The absolute differences between the ground-truth sinogram, Fig. 6 ▸(*f*), and the sinograms in Figs. 6 ▸(*g*)–6 ▸(*j*) are displayed in Figs. 6 ▸(*k*)–6 ▸(*n*). Brighter pixels indicate larger errors. As indicated in Fig. 6 ▸(*a*), each significant object projected a sine-wave locus in the sinogram. However, the key projection loci in both Figs. 6 ▸(*b*) and 6 ▸(*c*) were interfered by artefacts generated during the synthesis.

RU-Net was then applied to correct the artefacts of the synthesized sinograms; training the network on the *Drosophila* data set took 7 h. The sinograms synthesized by bicubic interpolation and *CyclicGen* were then input to the trained RU-Net model. Figures 6 ▸(*d*) and 6 ▸(*e*) present the sinograms synthesized by bicubic interpolation and *CyclicGen*, respectively, after artefact removal with RU-Net, and Figs. 5 ▸(*e*) and 5 ▸(*f*) present the corresponding reconstructed images. As indicated in Figs. 6 ▸(*m*) and 6 ▸(*n*), the incorporation of RU-Net yielded a clear decrease in the severity of errors in sinogram synthesis. As shown in Fig. 6 ▸(*n*), the sinograms synthesized by the proposed method had less severe errors than those synthesized by other methods.

The images produced with bicubic interpolation had the most severe streak and arc artefacts; by contrast, the results of the proposed method were similar to the ground-truth images. We quantitatively compared the quality of the reconstructed tomographic images using the metrics of peak signal-to-noise ratio (PSNR) and structural-similarity index measure (SSIM). Tables 1 ▸ and 2 ▸ present the comparison results and the computation time required for reconstructing each image set, respectively. Although the proposed method required the longest computation time, its reconstructed tomographic images had superior PSNR and SSIM to those of the other methods.

### 3.2. Mouse data set

**Figure 7:**
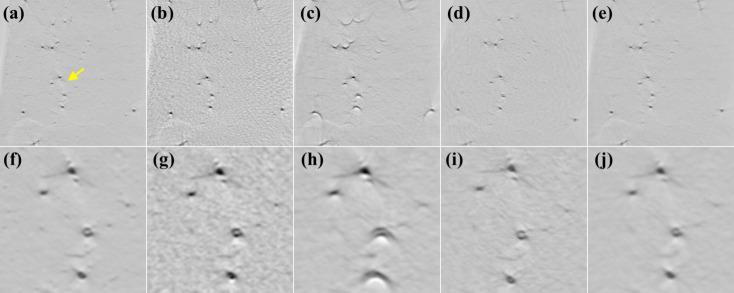
Tomographic images in the mouse data set. (*a*) The ground-truth image and images reconstructed from 75 projection views (*b*) directly and with the proposed method trained (*c*) on the mouse data set, (*d*) on the *Drosophila* data set or (*e*) with TL. (*f*)–(*j*) Magnifications of the region indicated by a yellow arrow in (*a*) for the corresponding figures in the first row.

Several studies on learning-based methods for SVCT have suggested that at least 25000 X-ray images should be used for training (Hu *et al.*, 2021 ▸; Chao *et al.*, 2022 ▸). However, collecting training data may be challenging, particularly for biological experiments for which sample preparation may take several days. In this experiment, we only collected 7800 X-ray images for the mouse data set. Golgi’s method was also applied for imaging the brain neurons of mice (Chin *et al.*, 2020 ▸). Fig. 7 ▸(*a*) displays a tomographic image in the mouse data set reconstructed from 600 projection views (the ground truth) and a magnification of the image is presented below in Fig. 7 ▸(*f*); images reconstructed with other methods are presented in the subfigures.

The proposed method was trained on the mouse training data set; however, *CyclicGen* produced results with SVCT noise and numerous artefacts [Fig. 7 ▸(*c*)].

We then applied the model trained on the larger *Drosophila* data set to reconstruct the mouse images. As shown in Fig. 7 ▸(*d*), the sinogram-synthesis artefacts were removed but the SVCT noise was still present. These results indicate that artefacts and noise may be generated by a model if the quantity of training data is insufficient. Therefore, we applied transfer learning (TL) (Pan & Yang, 2010 ▸) to reconstruct the mouse images. TL is performed as follows: for two data sets in two domains, **D**_1_ and **D**_2_ with **D**_1_ larger than **D**_2_, a learning model is first trained on **D**_1_; this model is called the pretrained model. The pretrained model can be further trained on **D**_2_ to refine its performance for the smaller domain. Hence, the proposed model trained on *Drosophila* (the pretrained model) was trained on the mouse data set. The tomographic image reconstructed through the proposed model after TL is presented in Fig. 7 ▸(*e*); Fig. 7 ▸(*j*) displays a magnified version of the image. Table 3 ▸ presents the average PSNR and SSIM of the reconstructed images; the columns FBP, +Mouse, +*Drosophila* and TL indicate the results for FBP without any correction, for the proposed method trained on the mouse data set, for the proposed method trained on the *Drosophila* data set and for the proposed method with TL, respectively. The experimental results reveal that the proposed model with TL had superior performance than the other models for the domain with insufficient training data.

## 4. Conclusions

We have developed a CNN approach based on *CyclicGen* and RU-Net for SXCT with sparse-view projections. In SVCT, streak artefacts and noise are often produced during sinogram reconstruction because the number of X-ray projections is insufficient – at fewer than 100 views. To address this problem, we employed *CyclicGen* to augment the X-ray projections, synthesized sinograms, and then applied RU-Net to correct synthesis errors in the produced sinograms. We validated the method on two data sets and demonstrated that it was effective for SXCT with sparse-view projections.

Specifically, the results indicate that tomographic images of 512 × 512 pixels can be reconstructed from 75 X-ray projections without visible streak artefacts or noise.

The proposed method can be used for a wide variety of applications in three-dimensional tomography. The artefacts and noise of sparse-view projections can be suppressed while preserving the main features if a sufficient amount of training data can be collected. Typically, training a model to reconstruct a volume of 512^3^ volumetric pixels requires ∼25000 training projection images. The process of obtaining training data for SXCT may also cause sample damage due to exposure to a high dose of radiation. However, TL can be used to first allow the model to learn artefact and noise patterns with sufficient training data collected from non-vulnerable objects or phantoms before being applied to the target domain. The proposed method can then effectively remove the streak artefacts and noise of sparse-view projections while preserving the appearance of the key objects in the images.

In further studies, we plan to improve the performance of the proposed model to a level where it is effective for fewer than 50 X-ray projections. Moreover, we intend to improve the model’s efficiency – specifically, to improve its ability to compensate for missing projections at a lower computational cost. We also intend to modify the proposed model to reconstruct only regions of interest from sparse-view projections. For example, a sparse-view projection reconstruction model could be used to reconstruct only the brain-neuron regions for the *Drosophlia* and mouse data sets.

## Figures and Tables

**Table 1:** PSNR and SSIM for the reconstructed *Drosophila* images

Bicubic		*CyclicGen*		Bicubic + RU-Net		*CyclicGen* + RU-Net	
PSNR	SSIM	PSNR	SSIM	PSNR	SSIM	PSNR	SSIM
48.61	0.990	50.28	0.993	49.40	0.993	54.64	0.997

**Table 2:** Time taken for image-set reconstruction

Bicubic	*CyclicGen*	Bicubic + RU-Net	*CyclicGen* + RU-Net
182 s	793 s	310 s	921 s

**Table 3:** Average PSNR and SSIM for the reconstructed mouse images

FBP		+Mouse		+*Drosophila*		TL	
PSNR	SSIM	PSNR	SSIM	PSNR	SSIM	PSNR	SSIM
21.07	0.493	22.45	0.747	39.89	0.934	42.85	0.949
